# *N*-Benzyl-2-(2,6-dichloro­phen­oxy)­acetamide

**DOI:** 10.1107/S1600536808022514

**Published:** 2008-07-26

**Authors:** Zhu-Bo Li, Yong-Huang Luo, Wen-Liang Dong, Jing Li, Hua Zuo

**Affiliations:** a College of Pharmaceutical Sciences, Southwest University, Chongqing 400716, People’s Republic of China; b Shandong University of Traditional Chinese Medicine, Jinan 250355, People’s Republic of China

## Abstract

The structure determination of the title compound, C_15_H_13_Cl_2_NO_2_, was undertaken as part of a project on the inter­action of small mol­ecules with proteins. In the crystal structure, the dihedral angle between the two aryl rings is 40.71 (11)°. The mol­ecules are connected *via* N—H⋯O hydrogen bonding into chains, which extend in the direction of the *b* axis.
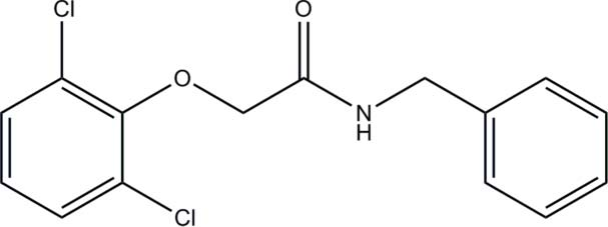

## Experimental

### 

#### Crystal data

C_15_H_13_Cl_2_NO_2_
*M* *_r_* = 310.16
Orthorhombic, 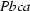

*a* = 14.8886 (10) Å
*b* = 8.6579 (6) Å
*c* = 22.9867 (14) Å
*V* = 2963.1 (3) Å^3^
*Z* = 8
Mo *K*α radiation
μ = 0.44 mm^−1^
*T* = 298 (2) K
0.20 × 0.20 × 0.10 mm

#### Data collection

Bruker APEXII CCD area-detector diffractometer
Absorption correction: multi-scan (*SADABS*; Bruker, 2005 ▶) *T*_min_ = 0.918, *T*_max_ = 0.958
16445 measured reflections
3412 independent reflections
2103 reflections with *I* > 2σ(*I*)
*R*_int_ = 0.040

#### Refinement

*R*[*F* ^2^ > 2σ(*F* ^2^)] = 0.044
*wR*(*F* ^2^) = 0.131
*S* = 1.02
3412 reflections
181 parameters
H-atom parameters constrained
Δρ_max_ = 0.20 e Å^−3^
Δρ_min_ = −0.34 e Å^−3^

### 

Data collection: *APEX2* (Bruker, 2005 ▶); cell refinement: *APEX2*; data reduction: *APEX2*; program(s) used to solve structure: *SIR97* (Altomare *et al.*, 1999 ▶); program(s) used to refine structure: *SHELXL97* (Sheldrick, 2008 ▶); molecular graphics: *SHELXTL* (Sheldrick, 2008 ▶); software used to prepare material for publication: *WinGX* (Farrugia, 1999 ▶).

## Supplementary Material

Crystal structure: contains datablocks I, global. DOI: 10.1107/S1600536808022514/nc2111sup1.cif

Structure factors: contains datablocks I. DOI: 10.1107/S1600536808022514/nc2111Isup2.hkl

Additional supplementary materials:  crystallographic information; 3D view; checkCIF report

## Figures and Tables

**Table 1:** Hydrogen-bond geometry (Å, °)

*D*—H⋯*A*	*D*—H	H⋯*A*	*D*⋯*A*	*D*—H⋯*A*
N1—H1*A*⋯O1	0.86	2.23	2.644 (2)	109
N1—H1*A*⋯O2^i^	0.86	2.31	2.970 (2)	133

Symmetry code: (i) 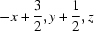.

**Fig. 1.**
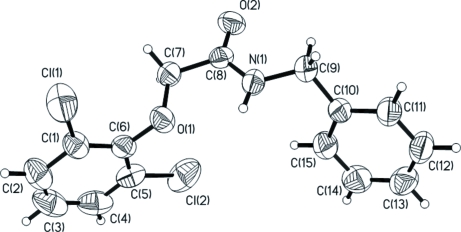
The molecular structure of the title compound with labelling and displacement ellipsoids drawn at the 50% probability level.

## supplementary crystallographic information

### Experimental

A solution of 2,6-dichlorophenol (1.0 mmol), *N*-benzyl-2-chloroacetamide (1.1 mmol), K_2_CO_3_ (1.1 mmol) in CH_3_CN (20 ml) was refluxed for 3 h and afterwards cooled down to room temperature. The solvent was removed under reduced pressure and the residue was poured into water and adjusted to pH 6–7. with dilute hydrochloric acid (10%) and extracted with ethyl acetate, washed with brine and dried over anhydrous MgSO_4_ to obtain the corresponding crude product. The product was obtained by column chromatography on silica gel using ethyl acetate as eluent. (yield 90%). Crystals suitable for X-ray diffraction were obtained by slow cooling of a solution of the solid in ethyl acetate/hexane at room temperature for 4 d.

### Refinement

All H atoms were placed in geometrically calculated positions and refined using a riding model with C—H = 0.97 Å (for CH_2_ groups) and 0.96 Å (for CH_3_ groups), their isotropic displacement parameters were set to 1.2 times (1.5 times for CH_3_ groups) the equivalent displacement parameter of their parent atoms.

### Crystal data

**Table:**

C_15_H_13_Cl_2_NO_2_	*D*_x_ = 1.391 Mg m^−^^3^
*M**_r_* = 310.16	Mo *K*α radiation λ = 0.71073 Å
Orthorhombic, *P**b**c**a*	Cell parameters from 2733 reflections
*a* = 14.8886 (10) Å	θ = 2.2–21.8º
*b* = 8.6579 (6) Å	µ = 0.44 mm^−^^1^
*c* = 22.9867 (14) Å	*T* = 298 (2) K
*V* = 2963.1 (3) Å^3^	Block, colorless
*Z* = 8	0.20 × 0.20 × 0.10 mm
*F*_000_ = 1280	

### Data collection

**Table:**

Bruker SMART CCD area-detector diffractometer	3412 independent reflections
Radiation source: fine-focus sealed tube	2103 reflections with *I* > 2σ(*I*)
Monochromator: graphite	*R*_int_ = 0.040
*T* = 298(2) K	θ_max_ = 27.5º
φ and ω scans	θ_min_ = 1.8º
Absorption correction: multi-scan(SADABS; Bruker, 2005)	*h* = −19→18
*T*_min_ = 0.918, *T*_max_ = 0.958	*k* = −10→11
16445 measured reflections	*l* = −20→29

### Refinement

**Table:**

Refinement on *F*^2^	Secondary atom site location: difference Fourier map
Least-squares matrix: full	Hydrogen site location: inferred from neighbouring sites
*R*[*F*^2^ > 2σ(*F*^2^)] = 0.045	H-atom parameters constrained
*wR*(*F*^2^) = 0.131	*w* = 1/[σ^2^(*F*_o_^2^) + (0.0631*P*)^2^ + 0.3666*P*] where *P* = (*F*_o_^2^ + 2*F*_c_^2^)/3
*S* = 1.02	(Δ/σ)_max_ = 0.001
3412 reflections	Δρ_max_ = 0.20 e Å^−^^3^
181 parameters	Δρ_min_ = −0.34 e Å^−^^3^
Primary atom site location: structure-invariant direct methods	Extinction correction: none

### Special details

**Table:**

Geometry. All e.s.d.'s (except the e.s.d. in the dihedral angle between two l.s. planes) are estimated using the full covariance matrix. The cell e.s.d.'s are taken into account individually in the estimation of e.s.d.'s in distances, angles and torsion angles; correlations between e.s.d.'s in cell parameters are only used when they are defined by crystal symmetry. An approximate (isotropic) treatment of cell e.s.d.'s is used for estimating e.s.d.'s involving l.s. planes.
Refinement. Refinement of *F*^2^ against ALL reflections. The weighted *R*-factor *wR* and goodness of fit *S* are based on *F*^2^, conventional *R*-factors *R* are based on *F*, with *F* set to zero for negative *F*^2^. The threshold expression of *F*^2^ > σ(*F*^2^) is used only for calculating *R*-factors(gt) *etc*. and is not relevant to the choice of reflections for refinement. *R*-factors based on *F*^2^ are statistically about twice as large as those based on *F*, and *R*- factors based on ALL data will be even larger.

### Fractional atomic coordinates and isotropic or equivalent isotropic displacement parameters (Å^2^)

**Table:**

	*x*	*y*	*z*	*U*_iso_*/*U*_eq_	
N1	0.74097 (11)	0.0194 (2)	0.64026 (7)	0.0521 (4)	
H1A	0.7004	0.0734	0.6230	0.063*	
Cl1	0.74730 (5)	0.13003 (7)	0.41952 (3)	0.0787 (2)	
Cl2	0.55214 (5)	−0.24316 (9)	0.55609 (4)	0.0985 (3)	
O1	0.68835 (9)	−0.00499 (15)	0.53078 (6)	0.0526 (4)	
O2	0.84330 (10)	−0.17088 (18)	0.62980 (6)	0.0591 (4)	
C1	0.65927 (14)	0.0023 (2)	0.42821 (9)	0.0515 (5)	
C2	0.60907 (17)	−0.0417 (3)	0.38036 (11)	0.0679 (7)	
H2A	0.6217	−0.0014	0.3438	0.081*	
C3	0.54023 (18)	−0.1457 (3)	0.38759 (14)	0.0802 (8)	
H3A	0.5057	−0.1746	0.3557	0.096*	
C4	0.52178 (17)	−0.2071 (3)	0.44101 (13)	0.0761 (8)	
H4A	0.4755	−0.2783	0.4454	0.091*	
C5	0.57263 (15)	−0.1625 (3)	0.48853 (11)	0.0603 (6)	
C6	0.64111 (13)	−0.0555 (2)	0.48288 (9)	0.0466 (5)	
C7	0.76294 (14)	−0.1008 (3)	0.54620 (9)	0.0522 (5)	
H7A	0.8148	−0.0717	0.5231	0.063*	
H7B	0.7487	−0.2076	0.5375	0.063*	
C8	0.78542 (13)	−0.0856 (2)	0.60971 (9)	0.0433 (5)	
C9	0.75781 (14)	0.0471 (3)	0.70161 (9)	0.0597 (6)	
H9A	0.7848	0.1484	0.7060	0.072*	
H9B	0.8007	−0.0286	0.7155	0.072*	
C10	0.67475 (13)	0.0393 (2)	0.73885 (8)	0.0471 (5)	
C11	0.67143 (16)	0.1244 (3)	0.78942 (9)	0.0586 (6)	
H11A	0.7190	0.1894	0.7988	0.070*	
C12	0.59859 (17)	0.1148 (3)	0.82645 (10)	0.0696 (7)	
H12A	0.5979	0.1718	0.8607	0.084*	
C13	0.52742 (17)	0.0216 (3)	0.81283 (11)	0.0693 (7)	
H13A	0.4780	0.0165	0.8375	0.083*	
C14	0.52928 (16)	−0.0638 (3)	0.76294 (11)	0.0697 (7)	
H14A	0.4814	−0.1283	0.7538	0.084*	
C15	0.60248 (15)	−0.0545 (3)	0.72586 (10)	0.0609 (6)	
H15A	0.6029	−0.1123	0.6918	0.073*	

### Atomic displacement parameters (Å^2^)

**Table:**

	*U*^11^	*U*^22^	*U*^33^	*U*^12^	*U*^13^	*U*^23^
N1	0.0540 (10)	0.0620 (11)	0.0404 (9)	0.0117 (8)	0.0022 (8)	−0.0039 (8)
Cl1	0.0982 (5)	0.0733 (4)	0.0644 (4)	−0.0303 (4)	−0.0038 (3)	0.0045 (3)
Cl2	0.0904 (5)	0.1033 (6)	0.1019 (6)	−0.0081 (4)	0.0394 (4)	0.0184 (4)
O1	0.0594 (9)	0.0521 (8)	0.0463 (8)	0.0140 (7)	−0.0084 (7)	−0.0094 (6)
O2	0.0484 (8)	0.0657 (9)	0.0633 (10)	0.0121 (7)	−0.0112 (7)	−0.0085 (7)
C1	0.0582 (12)	0.0460 (12)	0.0503 (12)	0.0008 (10)	−0.0087 (10)	−0.0080 (9)
C2	0.0867 (18)	0.0606 (14)	0.0565 (14)	0.0067 (13)	−0.0203 (12)	−0.0093 (11)
C3	0.0734 (18)	0.0740 (18)	0.093 (2)	0.0044 (14)	−0.0355 (16)	−0.0234 (15)
C4	0.0485 (13)	0.0675 (17)	0.112 (2)	−0.0034 (12)	−0.0089 (14)	−0.0132 (15)
C5	0.0466 (12)	0.0599 (13)	0.0745 (16)	0.0051 (11)	0.0084 (11)	−0.0035 (11)
C6	0.0443 (11)	0.0458 (11)	0.0497 (12)	0.0084 (9)	−0.0027 (9)	−0.0087 (9)
C7	0.0507 (12)	0.0603 (13)	0.0456 (12)	0.0127 (10)	0.0006 (9)	−0.0098 (9)
C8	0.0362 (10)	0.0448 (11)	0.0488 (12)	−0.0023 (9)	0.0028 (9)	−0.0019 (9)
C9	0.0522 (12)	0.0828 (17)	0.0441 (12)	−0.0021 (11)	0.0004 (10)	−0.0138 (11)
C10	0.0481 (11)	0.0523 (12)	0.0409 (11)	0.0042 (9)	−0.0021 (9)	0.0014 (9)
C11	0.0599 (13)	0.0665 (15)	0.0495 (12)	−0.0068 (11)	0.0043 (11)	−0.0085 (10)
C12	0.0749 (16)	0.0836 (17)	0.0503 (13)	−0.0039 (14)	0.0138 (12)	−0.0113 (12)
C13	0.0610 (14)	0.0832 (18)	0.0636 (16)	−0.0015 (13)	0.0164 (12)	0.0084 (13)
C14	0.0580 (14)	0.0770 (16)	0.0740 (17)	−0.0139 (13)	0.0017 (12)	0.0038 (13)
C15	0.0621 (14)	0.0654 (14)	0.0552 (14)	−0.0045 (12)	−0.0020 (11)	−0.0112 (11)

### Geometric parameters (Å, °)

**Table:**

N1—C8	1.326 (2)	C7—C8	1.504 (3)
N1—C9	1.452 (3)	C7—H7A	0.9700
N1—H1A	0.8600	C7—H7B	0.9700
Cl1—C1	1.726 (2)	C9—C10	1.506 (3)
Cl2—C5	1.730 (3)	C9—H9A	0.9700
O1—C6	1.378 (2)	C9—H9B	0.9700
O1—C7	1.431 (2)	C10—C11	1.378 (3)
O2—C8	1.225 (2)	C10—C15	1.381 (3)
C1—C6	1.379 (3)	C11—C12	1.381 (3)
C1—C2	1.383 (3)	C11—H11A	0.9300
C2—C3	1.374 (4)	C12—C13	1.368 (3)
C2—H2A	0.9300	C12—H12A	0.9300
C3—C4	1.366 (4)	C13—C14	1.365 (3)
C3—H3A	0.9300	C13—H13A	0.9300
C4—C5	1.384 (3)	C14—C15	1.386 (3)
C4—H4A	0.9300	C14—H14A	0.9300
C5—C6	1.384 (3)	C15—H15A	0.9300
			
C8—N1—C9	122.78 (18)	O2—C8—N1	124.39 (19)
C8—N1—H1A	118.6	O2—C8—C7	118.03 (18)
C9—N1—H1A	118.6	N1—C8—C7	117.58 (17)
C6—O1—C7	114.23 (14)	N1—C9—C10	113.75 (17)
C6—C1—C2	121.2 (2)	N1—C9—H9A	108.8
C6—C1—Cl1	119.12 (15)	C10—C9—H9A	108.8
C2—C1—Cl1	119.65 (18)	N1—C9—H9B	108.8
C3—C2—C1	119.2 (2)	C10—C9—H9B	108.8
C3—C2—H2A	120.4	H9A—C9—H9B	107.7
C1—C2—H2A	120.4	C11—C10—C15	118.0 (2)
C4—C3—C2	120.9 (2)	C11—C10—C9	119.02 (19)
C4—C3—H3A	119.5	C15—C10—C9	122.96 (19)
C2—C3—H3A	119.5	C10—C11—C12	121.1 (2)
C3—C4—C5	119.4 (2)	C10—C11—H11A	119.5
C3—C4—H4A	120.3	C12—C11—H11A	119.5
C5—C4—H4A	120.3	C13—C12—C11	120.2 (2)
C6—C5—C4	121.0 (2)	C13—C12—H12A	119.9
C6—C5—Cl2	118.98 (18)	C11—C12—H12A	119.9
C4—C5—Cl2	120.0 (2)	C14—C13—C12	119.7 (2)
O1—C6—C1	120.84 (19)	C14—C13—H13A	120.1
O1—C6—C5	120.90 (19)	C12—C13—H13A	120.1
C1—C6—C5	118.23 (19)	C13—C14—C15	120.1 (2)
O1—C7—C8	111.28 (15)	C13—C14—H14A	120.0
O1—C7—H7A	109.4	C15—C14—H14A	120.0
C8—C7—H7A	109.4	C10—C15—C14	120.9 (2)
O1—C7—H7B	109.4	C10—C15—H15A	119.5
C8—C7—H7B	109.4	C14—C15—H15A	119.5
H7A—C7—H7B	108.0		
			
C6—C1—C2—C3	−0.4 (3)	C6—O1—C7—C8	−154.72 (17)
Cl1—C1—C2—C3	178.76 (18)	C9—N1—C8—O2	1.3 (3)
C1—C2—C3—C4	−0.9 (4)	C9—N1—C8—C7	−178.76 (19)
C2—C3—C4—C5	0.7 (4)	O1—C7—C8—O2	174.13 (18)
C3—C4—C5—C6	0.7 (4)	O1—C7—C8—N1	−5.8 (3)
C3—C4—C5—Cl2	−178.2 (2)	C8—N1—C9—C10	−126.9 (2)
C7—O1—C6—C1	−95.4 (2)	N1—C9—C10—C11	−151.9 (2)
C7—O1—C6—C5	86.3 (2)	N1—C9—C10—C15	30.6 (3)
C2—C1—C6—O1	−176.57 (18)	C15—C10—C11—C12	0.9 (3)
Cl1—C1—C6—O1	4.3 (3)	C9—C10—C11—C12	−176.7 (2)
C2—C1—C6—C5	1.7 (3)	C10—C11—C12—C13	−1.1 (4)
Cl1—C1—C6—C5	−177.41 (15)	C11—C12—C13—C14	1.1 (4)
C4—C5—C6—O1	176.40 (19)	C12—C13—C14—C15	−0.9 (4)
Cl2—C5—C6—O1	−4.6 (3)	C11—C10—C15—C14	−0.7 (3)
C4—C5—C6—C1	−1.9 (3)	C9—C10—C15—C14	176.8 (2)
Cl2—C5—C6—C1	177.05 (16)	C13—C14—C15—C10	0.7 (4)

### Hydrogen-bond geometry (Å, °)

**Table:**

*D*—H···*A*	*D*—H	H···*A*	*D*···*A*	*D*—H···*A*
N1—H1A···O1	0.86	2.23	2.644 (2)	109
N1—H1A···O2^i^	0.86	2.31	2.970 (2)	133

Symmetry codes: (i) −*x*+3/2, *y*+1/2, *z*.
